# Identification of specific metabolic pathways as druggable targets regulating the sensitivity to cyanide poisoning

**DOI:** 10.1371/journal.pone.0193889

**Published:** 2018-06-07

**Authors:** Patrick Y. Sips, Xu Shi, Gabriel Musso, Anjali K. Nath, Yanbin Zhao, Jason Nielson, Jordan Morningstar, Amy E. Kelly, Brittney Mikell, Eva Buys, Vikhyat Bebarta, Jared Rutter, V. Jo Davisson, Sari Mahon, Matthew Brenner, Gerry R. Boss, Randall T. Peterson, Robert E. Gerszten, Calum A. MacRae

**Affiliations:** 1 Division of Cardiovascular Medicine, Department of Medicine, Brigham and Women’s Hospital and Harvard Medical School, Boston, United States of America; 2 Center for Medical Genetics Ghent, Department of Pediatrics and Medical Genetics, Ghent University, Ghent, Belgium; 3 Division of Cardiovascular Medicine, Beth Israel Deaconess Medical Center, Boston, United States of America; 4 Division of Cardiology, Department of Medicine, Cardiovascular Research Center, Massachusetts General Hospital, Charlestown, United States of America; 5 Broad Institute, Cambridge, United States of America; 6 Department of Pharmacology and Toxicology, College of Pharmacy, University of Utah, Salt Lake City, United States of America; 7 Department of Emergency Medicine, University of Colorado School of Medicine, Aurora, United States of America; 8 Department of Biochemistry and Howard Hughes Medical Institute, University of Utah, Salt Lake City, United States of America; 9 Department of Medicinal Chemistry and Molecular Pharmacology, Purdue College of Pharmacy, West Lafayette, United States of America; 10 Department of Medicine, Beckman Laser Institute, University of California, Irvine, United States of America; 11 Department of Medicine, University of California, San Diego, La Jolla, United States of America; Oregon State University, UNITED STATES

## Abstract

Cyanide is a potent toxic agent, and the few available antidotes are not amenable to rapid deployment in mass exposures. As a result, there are ongoing efforts to exploit different animal models to identify novel countermeasures. We have created a pipeline that combines high-throughput screening in zebrafish with subsequent validation in two mammalian small animal models as well as a porcine large animal model. We found that zebrafish embryos in the first 3 days post fertilization (dpf) are highly resistant to cyanide, becoming progressively more sensitive thereafter. Unbiased analysis of gene expression in response to several hours of ultimately lethal doses of cyanide in both 1 and 7 dpf zebrafish revealed modest changes in iron-related proteins associated with the age-dependent cyanide resistance. Metabolomics measurements demonstrated significant age-dependent differences in energy metabolism during cyanide exposure which prompted us to test modulators of the tricarboxylic acid cycle and related metabolic processes as potential antidotes. In cyanide-sensitive 7 dpf larvae, we identified several such compounds that offer significant protection against cyanide toxicity. Modulators of the pyruvate dehydrogenase complex, as well as the small molecule sodium glyoxylate, consistently protected against cyanide toxicity in 7 dpf zebrafish larvae. Together, our results indicate that the resistance of zebrafish embryos to cyanide toxicity during early development is related to an altered regulation of cellular metabolism, which we propose may be exploited as a potential target for the development of novel antidotes against cyanide poisoning.

## Introduction

Cyanide causes rapid toxicity upon exposure, in large part due to the inhibition of cytochrome c oxidase-dependent cellular respiration [1, 2], although other mechanisms are also likely involved owing to its reactive nature [3]. The acute clinical effects of cyanide are a direct function of cellular pseudohypoxia and acidosis due to the inability of cells to extract oxygen for respiration. Depending on the level of exposure, symptoms range from dizziness, headache, and hyperventilation to loss of consciousness, hemodynamic compromise, arrhythmias, seizures, apnea, cardiac arrest and finally death, which can occur rapidly with high concentrations of cyanide [4]. These symptoms reflect the particular vulnerability of excitable cells, both in the central nervous system as well as in the myocardium, to metabolic poisoning.

Exposure to cyanide can result from accidental spills, ingestion, smoke inhalation, or as an iatrogenic outcome, for example in the context of clinical sodium nitroprusside infusion [4]. Currently only a small number of antidotes are available which are limited in their clinical utility. These antidotes require intravenous administration, which in turn constrains the scale of deployment in the event of a mass casualty scenario. In addition, current antidotes are disadvantaged by several toxicities associated with their modes of action which limit their use[4–6]. There is an urgent need for novel, more effective cyanide countermeasures, which ideally could be deployed at scale by first responders in the field. This would not only provide measurable, life-saving benefits to the victims of small-scale poisonings, but would also have an impact in the face of a large-scale exposures as might occur during an industrial accident or a terrorist attack, where no practical options currently exist. In such an event, ready access to cyanide antidotes could transform a potentially devastating situation into a manageable one.

The currently available cyanide antidotes clear cyanide from the system either by sequestration or conversion to a less toxic form. More specifically, sodium thiosulfate relies on promoting cellular detoxification of cyanide by supplying additional substrate to the sulfur transferase enzyme system [7], while sodium nitrite enhances the circulating levels of methemoglobin which has a high affinity for cyanide. The clinical effectiveness of these agents to treat cyanide poisoning was discovered in the first half of the last century [8], leading to the development of combinations of these drugs as the standard treatments for patients exposed to cyanide. Another antidotal mechanism that is exploited in the clinic is the direct scavenging of free cyanide by cobalt-containing compounds, such as hydroxocobalamin (vitamin B12a) [9], dicobalt edetate [10], cobinamide [11], or related compounds [12], into inactive complexes. Similar agents leading to improved clearing of cyanide have been developed more recently as potential cyanide antidotes [13]. Notably, these agents are not very effective when used prophylactically or to aid in recovery, but they are of utility mainly when administered during the acute phase of cyanide poisoning.

We previously developed a zebrafish (*Danio rerio*) model as a first-line screening component of an integrated discovery pipeline for the identification and optimization of novel cyanide countermeasures [14, 15]. Recently, our collaborative efforts identified cisplatin analogs as a new class of cyanide antidotes demonstrating efficacy in zebrafish, mouse, and rabbit models [16]. These results highlight the relevance and translatability of the zebrafish model and demonstrate the potential for discovery of new cyanide countermeasures.

During our validation studies for the cyanide screening program in zebrafish embryos and larvae, we observed an age-dependent sensitivity to cyanide toxicity with progressive increases in the sensitivity during the first few days of development. In this study, we set out to investigate the mechanisms associated with this age-dependent effect as well as to discover drugs that could modulate these processes. Apart from improving our understanding of the fundamental biological processes associated with cyanide toxicity, the results described here also may lead to the development of novel cyanide antidotes.

## Materials and methods

### Zebrafish

In this study, zebrafish of a mixed Tübingen x AB background were used. Zebrafish husbandry and procedures performed in this study have been approved by the Harvard Medical Area Standing Committee on Animals, the local Institutional Animal Care and Use Committee (IACUC) which is certified by the Association for Assessment and Accreditation of Laboratory Animal Care (Protocol number: 04650). Zebrafish euthanasia was performed using an overdose of tricaine (3-aminobenzoic acid ethyl ester) in combination with hypothermic shock, according to the guidelines of the American Veterinary Medical Association. All personnel working with zebrafish received the appropriate training to handle zebrafish embryos, larvae, and adults, and to recognize behavior patterns related to pain or distress.

This study used death as an endpoint to evaluate the effects of cyanide administration to zebrafish embryos and larvae. Alternative endpoints could not be used in this study because larvae appear moribund after initial cyanide exposure, yet can still recover after treatment with an antidote. Therefore, premature euthanasia of cyanide-exposed zebrafish would preclude the identification of effective candidate compounds. Alternative models were not suitable since the experiments performed in this study require testing the response of a whole living organism to cyanide exposure. The use of a whole organism as an experimental model introduces the level of complexity needed to test the impact of cyanide and potential antidotes on vital integrative homeostatic functions including neuronal and cardiac physiology *in vivo*, which cannot be mimicked by experiments in cell culture. We chose to make use of zebrafish because they are the most tractable widely used vertebrate animal model, and, as a result of the available genetic and genomic resources and the high degree of physiologic representation, can provide results that are relevant for higher species including humans. The experiments in this study were performed on zebrafish embryos and larvae up to 8 dpf, avoiding unnecessary lethal exposures in fully developed adult animals which is likely to lead to increased levels of pain and distress.

The experiments with zebrafish embryos during the first days of development are not subject to animal welfare regulation, since zebrafish are not considered a protected animal species for scientific research until hatching at 72 hours post fertilization (hpf) according to the United States Public Health Service Policy on Humane Care and Use of Laboratory Animals, or until active feeding behavior develops (after 120 hpf) according to the European Union directive 2010/63/EU [17]. The local IACUC reviewed the proposed experiments on zebrafish larvae older than 72 hpf and approved the exposure to cyanide and other metabolic poisons in larvae up to 8 dpf. Zebrafish larvae were exposed to cyanide and routinely monitored hourly for the first 6h after exposure, and again at 24h, depending on the experimental setup. Zebrafish were scored as dead when prolonged cardiac arrest and/or tissue degradation was observed. All surviving fish were euthanized after 24h of exposure. For transcriptomics and metabolomics experiments, zebrafish samples were harvested after cyanide exposure for 6h or 2-3h, respectively. Dead fish were removed prior to further sample processing. In this study, a total of 2900 zebrafish larvae older than 72 hpf died after exposure to cyanide or another toxic agent, and an additional 265 were exposed to a lethal dose but were euthanized before reaching death as an endpoint.

### Drug administration

Potassium cyanide (KCN) as well as other drugs were administered to zebrafish embryos and larvae in E3 embryo medium (containing 5 mM NaCl, 0.17 mM KCl, 0.33 mM CaCl_2_, 0.33 mM MgSO_4_, and 10 mM HEPES pH 7.1) in 96-well plates for compound testing, and in 6-well dishes for gene expression analysis and metabolomic profiling experiments. In cases in which drugs were dissolved in DMSO, dilutions were made so the final DMSO concentration did not exceed 1% in the well, and the relevant vehicle control was used for each experiment.

### Gene expression analysis

Zebrafish embryos and larvae were harvested after 6h of exposure to vehicle or KCN at the indicated doses. Dead zebrafish were removed from the samples prior to processing. mRNA was extracted from pooled whole zebrafish embryos or larvae and hybridized to a GeneChip Zebrafish Genome Array (Affymetrix) and the raw data were processed and normalized as previously described [18]. Expression data were then mapped from Affymetrix Probeset IDs to Zebrafish Gene IDs using the Synergizer web tool [19]. Following mapping, expression data were averaged for each annotated gene, and then averaged across experimental replicates. 10,146 genes were ranked based on the fold change in average expression, as compared to vehicle treatment for a given time point. Heatmaps were visualized using the R statistical framework (www.r-project.org).

Next, genes were ranked by fold-change in different experimental conditions. These ranked datasets were then used as the input for gene set enrichment analysis (GSEA) [20]. GSEA pre-ranked analysis also requires a list of gene sets that will be examined for enrichment at the top or bottom of the ranked expression datasets. Gene Ontology (GO) [21] annotations were used for this purpose. GO SLIM biological process, molecular function, and cellular compartment annotations for zebrafish were downloaded from the BioMart community portal [22] and converted to GSEA-compatible GMT format using an in-house Perl script. The GSEA normalized enrichment scores are presented for processes with a false discovery rate q-value and a familywise-error rate p-value both smaller than 0.05.

### Metabolomics

Zebrafish embryos and larvae (50–100 per sample) were collected after exposure to cyanide or vehicle, washed 3 times in MilliQ water, and snap frozen in liquid N_2_. Whole zebrafish samples were thawed in MilliQ water and homogenized using a mortar and pestle on ice. Protein concentration was determined in the lysate using the Bradford assay, and further adjusted to 5 mg/ml with MilliQ water. A first set of 88 endogenous metabolites including tricarboxylic acid (TCA) intermediates, carbohydrates, and bile acids were extracted by adding 30 μl of the fish homogenate to 70 μl of acetonitrile/methanol (75:25; v:v) containing deuterated internal standards (25 μM thymine-d4 [Sigma-Aldrich], 10 μM inosine-15N4 [Cambridge Isotope Laboratories], 10 μM citrulline-d7 [Sigma-Aldrich], 25 μM phenylalanine-d8 [Cambridge Isotope Laboratories] and 10 μM valine-d8 [Sigma-Aldrich]). After vortexing, the samples were centrifuged at 20,000 g at 4°C for 20 min and supernatants were transferred to HPLC quality glass vials with inserts (MicroSolv). The multiple reaction monitoring-based LC-MS/MS method [23] was applied for metabolite profiling. In brief, 5 μl supernatant was loaded onto Xbridge Amide column (2.1×100mm 3.5 μm, Waters) coupled with 6490-QQQ iFunnel mass spectrometer in negative mode. The metabolites were separated with 1290 Infinity HLPC binary pump system (Agilent) using a gradient from 15% buffer A [water/acetonitrile (95:5, v:v) with 20 mM ammonium acetate and 20 mM ammonium hydroxide (pH 9.5)] and 85% buffer B (acetonitrile) to 65% buffer A and 35% buffer B in 6 minutes. The column compartment was maintained at 30°C. The eluted metabolites were measured by a coupled mass spectrometer in negative mode. The mass spectrometry settings for the QQQ 6490 were sheath gas temperature 400°C, sheath gas flow 12 L/min, drying gas temperature 290°C, drying gas flow 15 L/min, capillary 4000V, nozzle pressure 30 psi, nozzle voltage 500V and delta EMV 200V. Metabolite quantification was determined by integrating peak areas using MassHunter QQQ Quant (Agilent). All metabolite peaks were manually reviewed for peak quality in a blinded manner.

A second set of 48 metabolites including amino acids, vitamins, nucleotides and carnitines were profiled using previously described methods [24]. In brief, 10 μl of fish lysate was added into the extraction buffer to precipitate the protein, and 10 μl of supernatant was loaded onto a hydrophilic interaction chromatography column for separation. The eluent was further detected by a coupled 4000 QTRAP mass spectrometer (ABSciex) in the positive mode. Metabolite peaks were integrated using MultiQuant software (ABSciex).

Metabolomics data were analyzed using the online MetaboAnalyst 3.5 tool (http://www.metaboanalyst.ca) as described [25, 26]. Briefly, to compare responses to cyanide challenge, fold changes for different metabolite levels before and after cyanide were ranked and the top- and bottom-ranked metabolites were used as input for over-representation analysis using the hypergeometric test in the Metabolic pathway analysis module. To compare the baseline between 1 dpf embryos and 7 dpf larvae, metabolite concentrations were input and analyzed using the Globaltest pathway enrichment analysis method. The input data were mapped to the zebrafish pathway library, and for both datasets pathway topology analysis was performed using relative betweenness centrality as the node importance measure. The MetaboAnalyst tool then ranks relevant pathways based on the p value from the pathway enrichment analysis and the impact value calculated from the pathway topology analysis.

### Morpholino knockdown

Morpholino antisense oligos (Gene Tools) were designed to block translation of zebrafish isoforms of PDK by interfering with ribosome binding to the translation start site. The sequences of the ATG-targeted morpholinos were AAGTCCTGAAGATCCTCATGTTGGC (PDK1) and CTAACAAACTTCATCTTGGAAAGCT (PDK2a). The morpholinos were resuspended in sterile water to a concentration of 1 mM. After further dilution in Danieau’s solution (58 mM NaCl, 0.7 mM KCl, 0.4 mM MgSO_4_, 0.6 mM Ca(NO_3_)_2_, 5 mM HEPES) to the desired concentration, 1 nl was injected into fertilized eggs at the single cell stage using an Eppendorff FemtoJet. After injection, the embryos were kept at 28°C in E3 solution.

## Results

### Age-dependent cyanide toxicity

**Fig 1:**
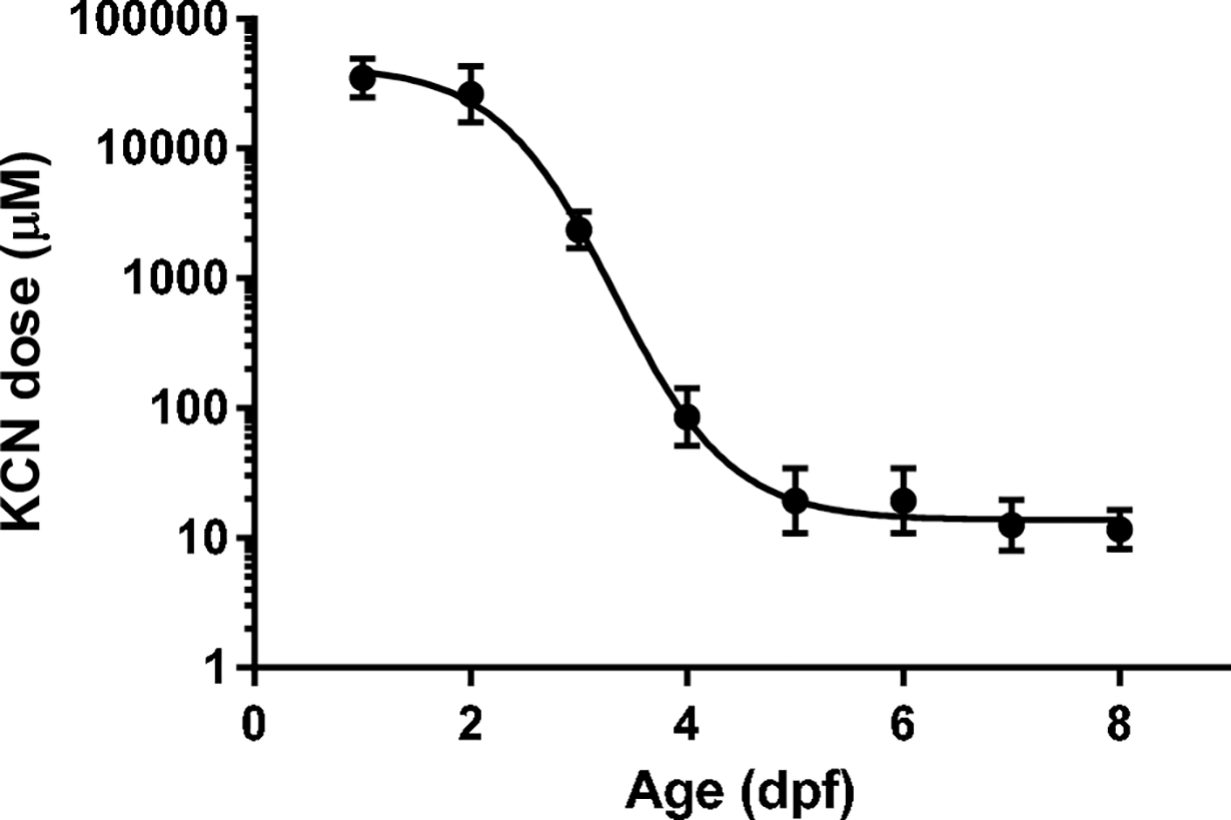
Sensitivity of zebrafish to cyanide depends on the developmental stage. The LD_50_ for 3h exposure to KCN in zebrafish is shown as a function of the developmental stage in days post fertilization (dpf). The best-fit sigmoidal curve calculated by non-linear regression analysis is plotted in the figure. LD_50_ values were calculated by non-linear regression fitting of dose-response curves showing survival after cyanide exposure in zebrafish embryos and larvae at different stages (S1 Dataset).

During the validation of our zebrafish model for cyanide toxicity, we observed that the resistance to KCN poisoning was significantly higher during the first days of development, gradually decreasing with age until reaching a plateau level after 5 dpf (Fig 1, S1 Dataset). 1–2 dpf embryos exposed to high sub-lethal doses of cyanide (50–100 μM KCN) demonstrated an arrested development, which lasted until the KCN was washed out or escaped from the aqueous medium (after approximately 1 day of incubation). At that point, embryos resumed their normal development and, apart from the developmental delay, did not show any further evidence of the prior cyanide exposure. KCN-induced lethality at higher doses in these young embryos was manifested as general progressive cell death and tissue damage, culminating in total disintegration of the embryo. Older larvae exposed to toxic doses of KCN (≥ 25 μM, depending on the specific stage) quickly showed signs of morbidity: lateral decubitus position, bradycardia, gasping, and lack of spontaneous or stimulus-induced movement. Mortality was identified as cardiac arrest, followed by tissue degradation. Interestingly, a similar age-dependent resistance was observed for two other metabolic poisons, sodium azide (NaN_3_) and hydrogen sulfide (H_2_S) (S1 Fig). However, the difference in lethal dose for these poisons between young (1 dpf) embryos and older (8 dpf) larvae was smaller (approximately 2 log orders) than for KCN (more than 3 log orders).

### Gene expression after cyanide exposure

**Fig 2:**
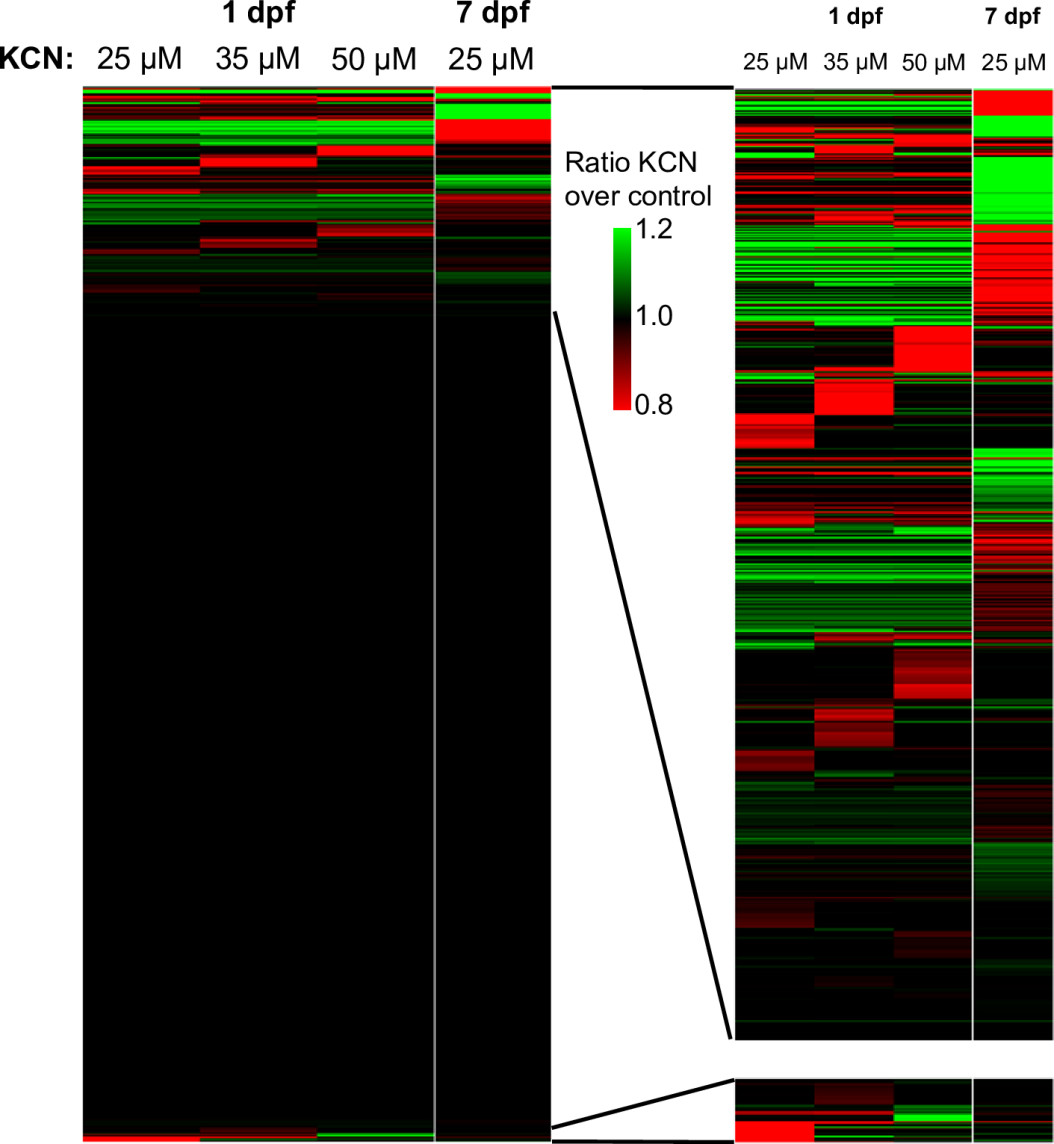
The transcriptional response to cyanide exposure is very limited in zebrafish embryos and larvae. Data from a microarray experiment comparing 1 dpf embryos and 7 dpf larvae exposed to KCN at the indicated doses was used to generate a heatmap showing expression changes of 10,146 genes as compared to the respective vehicle treatment. On the right an enlargement is shown of the clustered regions that show differential expression at the top and the bottom of the range.

We performed microarray-based expression profiling to identify potential molecular mechanisms associated with the age-dependent differences in sensitivity to cyanide. 7 dpf larvae were exposed to 25 μM KCN (lethal dose for this age) or vehicle, and 1 dpf embryos were exposed to 25, 35, or 50 μM KCN to identify expression patterns that are associated with the level of protection against cyanide toxicity. Our data demonstrated only very modest changes in gene expression after cyanide exposure (Fig 2). The largest magnitude of gene expression change was 20% in the comparison of 7 dpf larvae (8 genes had a 15–20% change from baseline), and only 10% in the 1 dpf samples. GSEA analysis of the expression data showed that KCN-induced changes were highly consistent between the different experimental groups (Table 1). The only exception was the serine-type endopeptidase activity, which was positively enriched in 1 dpf embryos exposed to 50 μM KCN but negatively enriched in 7 dpf larvae exposed to 25 μM KCN. Nevertheless, the enrichment of this process relies on identical, very subtle expression changes in 27 paralogues and pseudogenes of *HtrA2/Omi*, a consequence of an analysis artifact due to one cDNA probe mapping to these different genomic loci.

**Table 1:** Gene expression changes after exposure to cyanide at different ages.

GSEA Normalized Enrichment Scores	1 dpf			7 dpf	Control
Process	25 μM KCN vs control	30 μM KCN vs control	50 μM KCN vs control	25 μM KCN vs control	7 dpf vs 1 dpf
carbohydrate binding		-3.9	-3.9		
catalytic activity					3.16
cellular iron ion homeostasis	-2.9	-2.66	-3.01	-2.51	-3.04
ferric iron binding	-2.97	-2.63	-2.9	-2.54	-2.94
hemoglobin complex	-2.54	-2.57			
hydrolase activity	3.32	3.17	3.15	3.01	
kinase activity	3.36	3.09	2.85		
metabolic process	3.59	3.52	3.38	2.93	
multicellular organismal development	2.74	2.57	2.7		
phosphorylation	3.42	3.07	2.84		
proteasome complex					2.51
protein dimerization activity	2.86	2.85	2.87	2.71	
sequence-specific DNA binding	2.64	2.78	2.57		
sequence-specific DNA binding transcription factor activity	3.13	3.09	3		
serine-type endopeptidase activity			3.7	-3.09	2.92
transcription, DNA-dependent	3.61	3.65	3.52	2.71	2.67
transferase activity	2.84	2.64			
transition metal ion binding	-2.67	-2.92			-2.7
transport	2.62	2.54			
viral envelope		2.73	2.57	2.71	-2.73
viral reproduction	-2.6	-2.59	-2.57		

List of biological processes enriched in 1 dpf zebrafish embryos and 7 dpf larvae after 6h exposure to KCN at the indicated doses or at baseline, as identified from GSEA analysis of the microarray data presented in Fig 2. The GSEA normalized enrichment score indicates the relative direction and magnitude of pathway enrichment after cyanide exposure.

GSEA analysis was also performed to compare the basal transcriptomic profiles of 1 dpf vs 7 dpf zebrafish, to attempt to uncover protective gene expression patterns underlying the differences in sensitivity to cyanide. We found that only a few select processes were regulated in a significantly different manner between the two groups. Apart from some general terms related to ongoing tissue differentiation and growth, we found that multiple processes related to iron handling were significantly downregulated in 7 dpf vs 1 dpf zebrafish. The enrichment of the cellular iron ion homeostasis, ferric iron binding, and transition metal ion binding processes relies on the lower expression of genes involved in ferritin biosynthesis in 7 dpf larvae vs 1 dpf embryos. It is conceivable that this is linked to the increased use of cellular iron in various metabolic enzymes responsible for oxygen handling in 7 dpf larvae. The enrichment of the viral envelope process is related to the increased expression of genes encoding zona pellucida glycoproteins in 1 dpf embryos, which are the major components of the chorion surrounding oocytes and early embryos. Finally, as noted earlier the enrichment of the serine-type endopeptidase activity process we observed is due to an analysis artifact related to the HtrA2/Omi probe.

### Metabolomics after cyanide exposure

**Fig 3:**
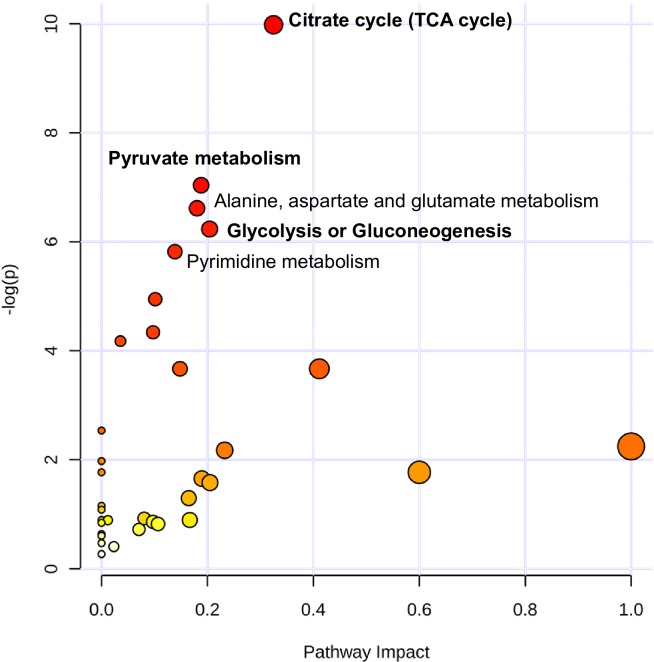
Comparison of the metabolomic responses of 1 dpf and 7 dpf zebrafish embryos to cyanide exposure. Results from the metabolomic analysis of 1 dpf and 7 dpf zebrafish embryos exposed to 500 μM or 20 μM KCN, respectively, or vehicle for 2 h. Pathway enrichment analysis results are plotted according to p-values and pathway impact scores. Pathways whose regulation in the response to cyanide is significantly different between different ages after correction for the false discovery rate are indicated in the figure; pathways related to energy metabolism are marked in boldface type.

**Fig 4:**
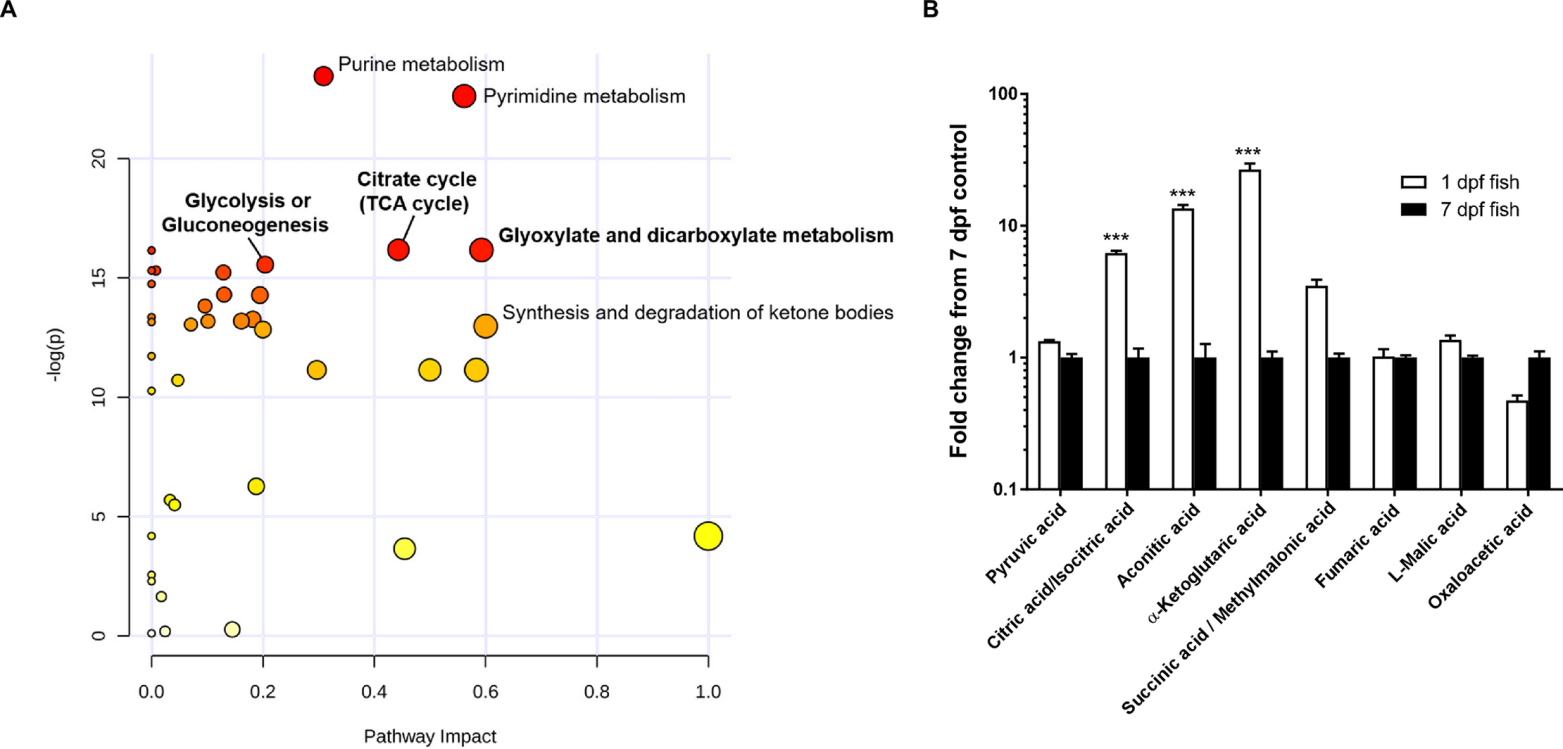
Comparison of the metabolomic signature of 1 dpf and 7 dpf zebrafish. A) Representation of the pathway enrichment analysis of the metabolite concentration levels at baseline in 1 dpf embryos vs 7 dpf larvae. Pathways are plotted according to p-values and pathway impact scores. The top significant pathways are indicated in the figure; pathways related to energy metabolism are in boldface type. B) Measurements of levels of TCA cycle intermediates in 1 and 7 dpf zebrafish. ***: P<0.001 vs 7 dpf larvae by Sidak’s multiple comparison test after two-way ANOVA.

To complement the gene expression studies, we assessed the metabolomic profiles of 1 dpf and 7 dpf zebrafish before and after KCN exposure. Metabolomics enables the unbiased detection of non-genomic changes in enzymatic activity of a range of cellular and metabolic processes. Metabolic pathway enrichment analysis revealed that the response to KCN was significantly different in 1 dpf vs. 7 dpf zebrafish with regard to energy metabolism-related processes involving the tricarboxylic acid (TCA) cycle, pyruvate metabolism, and glycolysis / gluconeogenesis (Fig 3, S1 Table). This prompted us to further examine the baseline metabolic differences between 1 dpf embryos and 7 dpf larvae prior to KCN challenge. Metabolomics profiling confirmed that, apart from differences in nucleotide and amino acid metabolism which are likely related to organism growth, several major metabolic pathways were significantly different between 1 dpf and 7 dpf zebrafish. In particular the TCA cycle, glyoxylate and dicarboxylate metabolism, and glycolysis / gluconeogenesis were highly significantly enriched processes with a high pathway topological impact (Fig 4A, S2 Table). Interestingly, we found that the levels of the TCA cycle intermediates citrate, aconitate, and α-ketoglutarate were significantly higher in 1 dpf embryos than in 7 dpf larvae (Fig 4B). These data suggest that fundamental differences in metabolism may underlie the observed age dependence of cyanide sensitivity.

### Focused screening of modulators of metabolism

**Fig 5:**
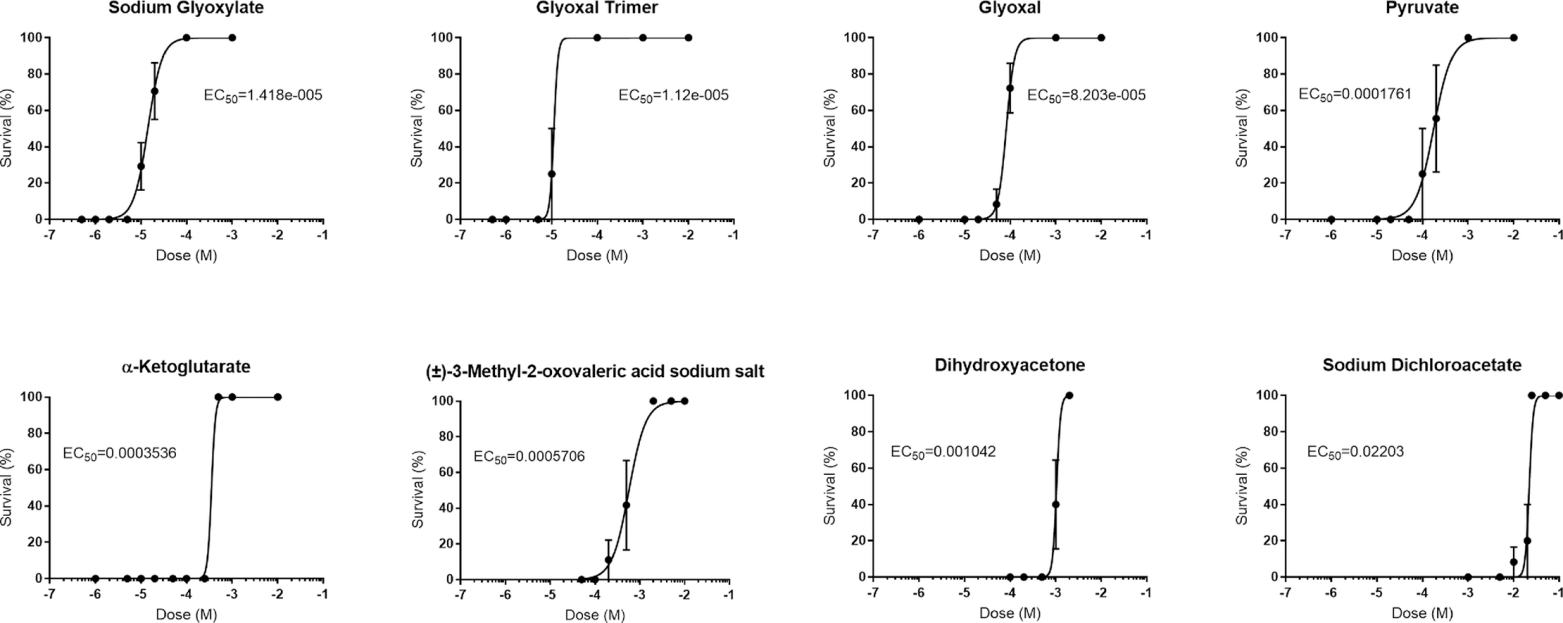
Hit compounds providing protection against cyanide toxicity. Survival of 7 dpf zebrafish larvae exposed to a lethal dose of 20 μM cyanide for 24h, in combination with the 8 compounds that were found to be protective in our focused drug screen. EC50 values were calculated by linear regression fitting of sigmoidal dose-response curves to the data (S4 Dataset).

In light of the evidence that differences in the TCA cycle and related cellular metabolic processes are associated with the observed age-dependent difference in sensitivity to cyanide, we performed a focused screen of small molecules, all of which had previously been annotated as modulators of energy metabolism, as cyanide antidotes. We tested the efficacy of 48 compounds at multiple doses for their ability to either prevent cyanide toxicity in 7 dpf zebrafish larvae or abrogate the resistance to KCN in 1 dpf embryos (S3 Table). Using this approach, we identified 8 drugs that, at their respective effective dose, completely rescued 7 dpf larvae from exposure to a lethal KCN dose, characterized by normal motility and response to stimuli (Fig 5, S4 Dataset). We also found 3 compounds that increased the sensitivity to KCN in 1 dpf embryos. The effective drugs could generally be classified as glyoxylate-related compounds or drugs targeting mitochondrial metabolic pathways associated with the TCA cycle, including the TCA metabolite α-ketoglutarate, the energy carrier sodium pyruvate, the α-ketoglutarate dehydrogenase inhibitor (±)-3-methyl-2-oxovaleric acid sodium salt, the glycolysis product dihydroxyacetone, and the pyruvate dehydrogenase kinase (PDK) inhibitor sodium dichloroacetate (DCA).

### Increased resistance to cyanide by modulation of the pyruvate dehydrogenase complex

**Fig 6:**
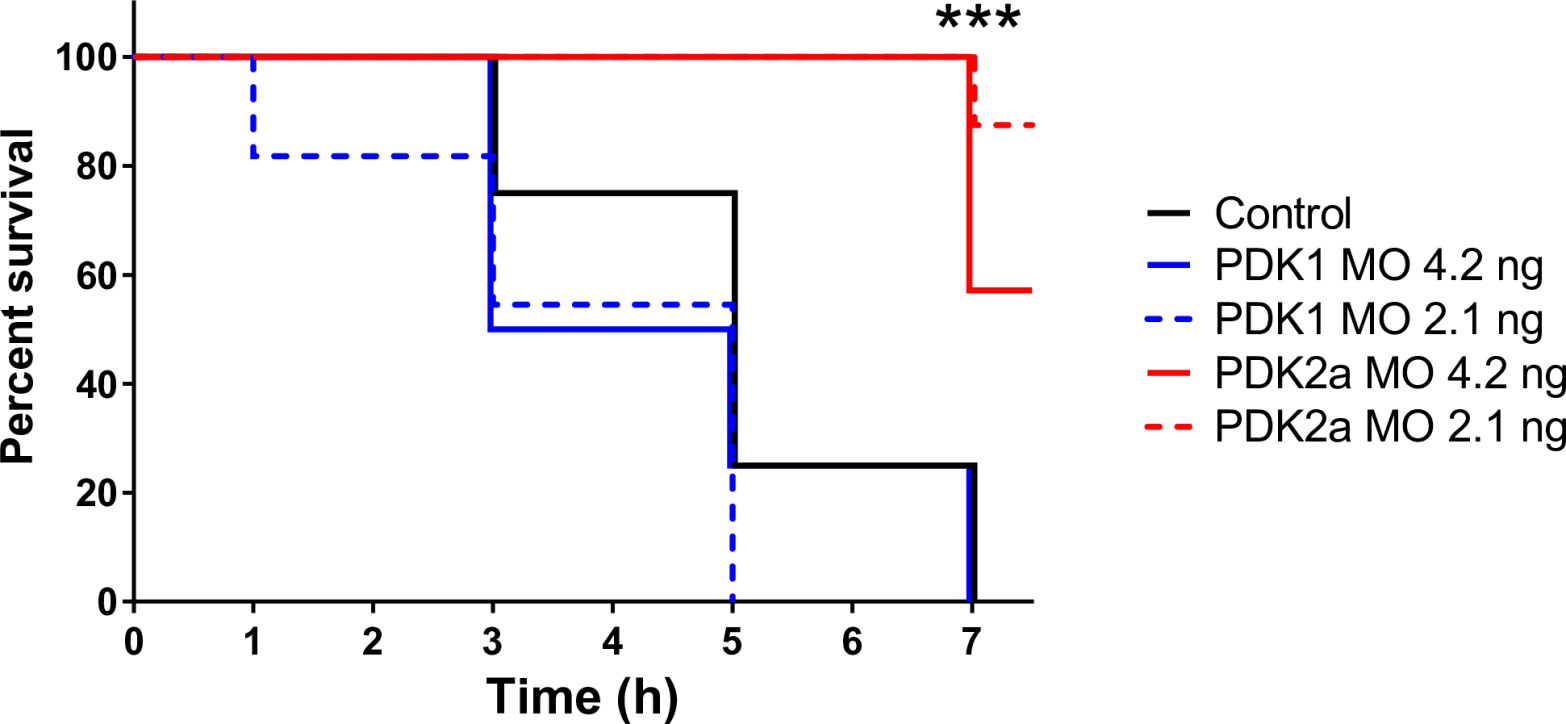
Pyruvate dehydrogenase kinase regulates sensitivity to cyanide. Survival curves of 4 dpf zebrafish control larvae and larvae injected with a synthetic morpholino (MO) targeting PDK1 or PDK2a after challenge with 100 μM KCN. N = 7–11 larvae per group. ***: P<0.001 by Log-rank (Mantel-Cox) test.

Considering the protective effects of pyruvate and DCA during cyanide exposure, we further investigated the role of PDK in sensitivity to KCN. Inhibition of PDK by DCA is expected to stimulate the pyruvate dehydrogenase complex, leading to increased flux of pyruvate into the mitochondria as substrate for the TCA cycle. We tested the effects of morpholino knockdown of zebrafish *pdk1* and *pdk2a*, coding for PDK isoforms that are known to be expressed during development, on sensitivity to KCN. Since the effects of morpholino knockdown would be diluted out after 7 days of development, we investigated the effects of PDK knockdown in 4 dpf larvae challenged with a lethal 100 μM KCN dose. We found that knockdown of *pdk2a*, but not *pdk1*, could improve resistance to KCN (Fig 6), confirming a potential role for this protective mechanism. The pdk2a knockdown embryos which survived during the 8-hour observation window post-cyanide challenge, died however at 5 dpf after 24h of cyanide exposure. This may however be due to the lack of morpholino efficacy at that stage.

### Protection against KCN toxicity by glyoxylate is associated with altered metabolism

**Fig 7:**
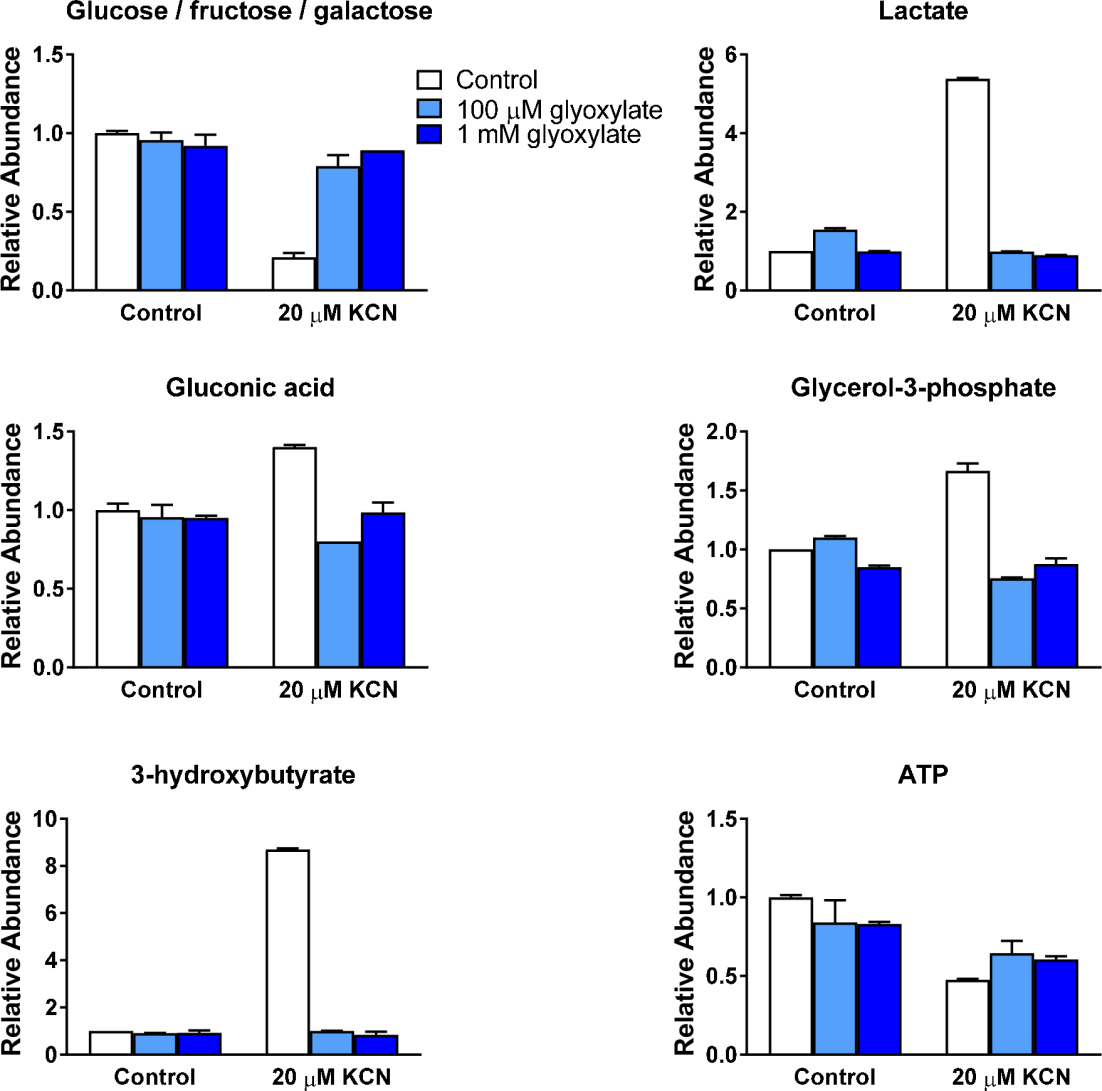
Glyoxylate reverses changes in cellular metabolism induced by cyanide. Selected metabolite levels were measured with and without KCN administration in pooled 7 dpf zebrafish larvae treated with sodium glyoxylate or vehicle. Data from replicate measurements are normalized to the level measured in 7 dpf control larvae. Increased levels of glucose and decreased levels of lactate, gluconic acid, and glycerol-3-phosphate indicate that glyoxylate treatment reduced KCN-induced glucose oxidation. Reduction of KCN-induced 3-hydroxybutyrate levels reflects a decreased level of ketosis during glyoxylate treatment. Glyoxylate treatment also leads to a mild improvement in ATP levels after KCN administration.

A key finding in our focused screen was that glyoxylate and its precursors glyoxal and glyoxal trimer were potent cyanide antidotes. These compounds could potentially function as substrates for the glyoxylate cycle, which operates as an alternative shunt in the TCA cycle. To further investigate the effects of glyoxylate, we performed metabolomic measurements after administration of sodium glyoxylate with or without KCN in 7 dpf larvae (Fig 7). These measurements suggested that multiple markers of cellular metabolism were improved after glyoxylate treatment, including a decrease in ketosis.

## Discussion

In the work we have outlined, we demonstrate that there is an age-dependent sensitivity to cyanide toxicity in zebrafish, which is associated with differences in the baseline cellular metabolic state. A deeper understanding of these developmental changes could potentially be leveraged to generate novel therapies for metabolic poisoning in adult organisms, and impact not only the treatment of cyanide exposure, but also treatment of other poisons or ischemic tissue injury. Interestingly, the effects of cyanide administration in zebrafish embryos are very similar to the effects observed during anoxia [27], where young embryos (before 2 dpf) transition to a state of arrested development, but can survive the anoxic challenge for an extended period of time. Several inhibitors of mitochondrial oxidative phosphorylation were shown to have similar effects, suggesting that this state of physiologic arrest is induced upon inhibition of aerobic respiration in early embryos. It has been suggested from *in vitro* data that AMP-activated protein kinase (AMPK) activation plays a role in the sensing of these metabolic perturbations [27]. Nevertheless, we did not find that AMPK-targeting drugs could provide any protection to cyanide in older zebrafish larvae.

In order to better understand the mechanisms associated with the age-dependent sensitivity to cyanide, we first performed an unbiased transcriptomic analysis between the resistant and sensitive stages of zebrafish development. Our results showed that the effects of cyanide on transcription are so profound that even in animals with sub-lethal exposure, there is only a minimal transcriptional response. Unbiased gene set enrichment analysis showed that within the very subtle changes in gene expression, only one process was found to be regulated differently between 1 dpf and 7 dpf zebrafish: the serine-type endopeptidase activity. Within this gene set, genes coding for HtrA2/Omi peptidase activity are enriched, although this is likely a result of artefactual probe mapping. Nevertheless, it is interesting to note that HtrA2/Omi is involved in the regulation of mitochondrial metabolism including the TCA cycle [28].

The finding that individual genes involved in iron ion handling, and more specifically genes encoding ferritin subunits, are expressed at higher levels in 1 dpf than 7 dpf zebrafish at baseline suggests that more free iron is available in early embryos. We hypothesize that as iron is incorporated in metal-containing proteins in 7 dpf larvae, there is a secondary downregulation of ferritin expression. This is likely a consequence of the transition of zebrafish embryos to an oxygen-based metabolism, where proteins responsible for electron transport, oxygen binding, and various other oxidative processes all fix iron. This evolutionarily conserved switch would also render the older organisms more sensitive to cyanide toxicity, as a result of the strong affinity of cyanide for metal ions.

The lack of a robust transcriptional response strongly suggests that the variation in susceptibility to cyanide is a consequence of the basal transcriptional state and thus non-genomic differential responses. Using metabolomics profiling, we have demonstrated substantial differences in the equilibrium state of the TCA cycle that are associated with discrete levels of cyanide resistance at different ages in zebrafish. In a subsequent focused small molecule screening approach, we were able to implicate PDK as a potential regulator of these differences. A genetic knockdown experiment further confirmed that modulation of the flux through the TCA cycle regulated by PDK is able to mitigate the effects of cyanide toxicity through unknown mechanisms.

Our chemical screen also identified sodium glyoxylate and several of its precursors as potent cyanide antidotes. However, a potential issue with these glyoxylate-related small molecules is that these compounds might also protect via the direct binding of cyanide to aldehydes and ketones, leading to cyanohydrin formation [29]. Nevertheless, we found that related control compounds with comparable reducing capacity did not exhibit any protective activity at similar doses, arguing for the hypothesis that the observed protection is largely a consequence of manipulation of the cellular metabolic state. This is further supported by the molar ratios of the minimum effective glyoxylate dose to the cyanide challenge in zebrafish larvae (glyoxylate: cyanide ratio ~1:1).

An interesting question is how glyoxylate exerts its protective effects against cyanide toxicity *in vivo*. It is conceivable that glyoxylate functions as a substrate for the glyoxylate cycle, which has been identified in plants and microorganisms as an alternative shunt in the TCA cycle bypassing two oxidative decarboxylation steps. This would allow gluconeogenesis from fatty acid substrate, which might supply essential energy fuel to the central nervous system, a primary target organ for cyanide toxicity. This phenomenon might also explain the decreased ketosis observed after glyoxylate administration during KCN exposure in zebrafish larvae. The glyoxylate cycle requires malate synthase activity, which catalyzes the conversion of glyoxylate and acetyl-coenzyme A (CoA) to malate and CoA, which are then further processed in the TCA cycle. While an enzyme coding for malate synthase is clearly present in the zebrafish genome, the orthologous gene in placental mammals appears to be a pseudogene, although other enzymes may have taken over its function [30, 31].

In summary, our study has identified metabolic processes associated with the TCA cycle and the pyruvate dehydrogenase complex as potential mechanisms for the age-dependent resistance to cyanide toxicity in zebrafish. Additionally, the small molecule glyoxylate was identified as a novel potent cyanide antidote. Further mechanistic and pharmacologic studies may lead to the development of new therapeutic strategies to treat metabolic poisoning.

## Supporting information

S1 Fig Age-dependent sensitivity to metabolic poisons in zebrafish. The LD_50_ for 3h exposure of zebrafish embryos and larvae to Na_2_S or NaN_3_ is shown as a function of the developmental age in days post fertilization (dpf). The best-fit sigmoidal curves, calculated by non-linear regression analysis, are plotted on the figure.
(TIF)

S1 Table Metabolic pathways differently regulated between zebrafish of different age after exposure to cyanide. Results of the pathway over-representation analysis of the metabolomics data comparing the KCN-induced metabolite response in 1 dpf embryos vs 7 dpf larvae using the online MetaboAnalyst 3.5 Metabolic pathway analysis module. The *Danio rerio* Kyoto Encyclopedia of Genes and Genomes (KEGG) identification number is given for each metabolic pathway. The total number of compounds, the random expected hits and the actually matched number from the user uploaded data (hits) are indicated for each pathway. The nominal p-value is calculated from the enrichment analysis, and the False Discovery Rate p is the p-value adjusted using the false discovery rate for the experiment. The Topology impact parameter is the pathway impact value calculated from pathway topology analysis.
(DOCX)

S2 Table Differences in metabolic signature at baseline between 1 and 7 dpf zebrafish. Results of the pathway enrichment analysis of the metabolomics data comparing baseline metabolite concentration levels in 1 dpf embryos vs 7 dpf larvae using the online MetaboAnalyst 3.5 Metabolic pathway analysis module. Parameters shown are as in S1 Table.
(DOCX)

S3 Table Compounds tested in the focused zebrafish screen of metabolism regulating drugs. EC50 doses calculated from 24h survival data of 7 dpf zebrafish larvae exposed to 20 μM KCN (S1 Dataset), as well as the EC50 dose for mortality in 1 dpf zebrafish embryos exposed to 500 μM KCN are shown, where applicable. Doses of the experimental drug (without KCN exposure) leading to zebrafish mortality after 24h exposure are shown as the relevant toxic dose of the drug. ND: not done; -: no effective dose found within range tested (the dose ranges went up to the maximum solubility of the respective drug in the embryo medium).
(DOCX)

S1 Dataset Survival data of zebrafish embryos and larvae after cyanide exposure. Raw data showing survival of zebrafish embryos and larvae at different ages after exposure to a dose range of KCN for 3h. The LD50 doses were calculated using non-linear regression of log-transformed KCN doses vs. survival in GraphPad Prism version 7.
(XLS)

S2 Dataset Ratios of metabolites measured in 1 dpf and 7 dpf zebrafish exposed to cyanide. Raw data of the ratio of the response of each metabolite level after cyanide exposure in 1 dpf embryos compared to 7 dpf larvae. Zebrafish were exposed to KCN for 2h prior to harvesting, at a dose of 500 μM KCN for 1 dpf embryos and 20 μM KCN for 7 dpf larvae.
(XLS)

S3 Dataset Metabolite concentrations used for comparison of the baseline metabolomic profile in 1 dpf and 7 dpf zebrafish. Raw data file showing the sample name and group, and listing the concentration of each metabolite (identified by its HMDB ID) as measured in our metabolomics platform. These data were input into the online MetaboAnalyst 3.5 tool for pathway enrichment analysis of differentially regulated processes at baseline in 1 dpf embryos vs 7 dpf larvae.
(XLS)

S4 Dataset Survival data of zebrafish larvae after cyanide exposure with hit compounds. Raw dose-response data of 7 dpf zebrafish larvae exposed for 24h to 20 μM KCN together with the indicated dose of the hit compound being tested. Each column of survival data represents a separate experiment (N = 3–8 zebrafish per dose per experiment).
(XLS)
