# Supplementary figures and images for: Identification of specific metabolic pathways as druggable targets regulating the sensitivity to cyanide poisoning
Source: PLoS One. 2018 Jun 7;13(6):e0193889. doi: 10.1371/journal.pone.0193889 (PMC5991913; doi:10.1371/journal.pone.0193889)

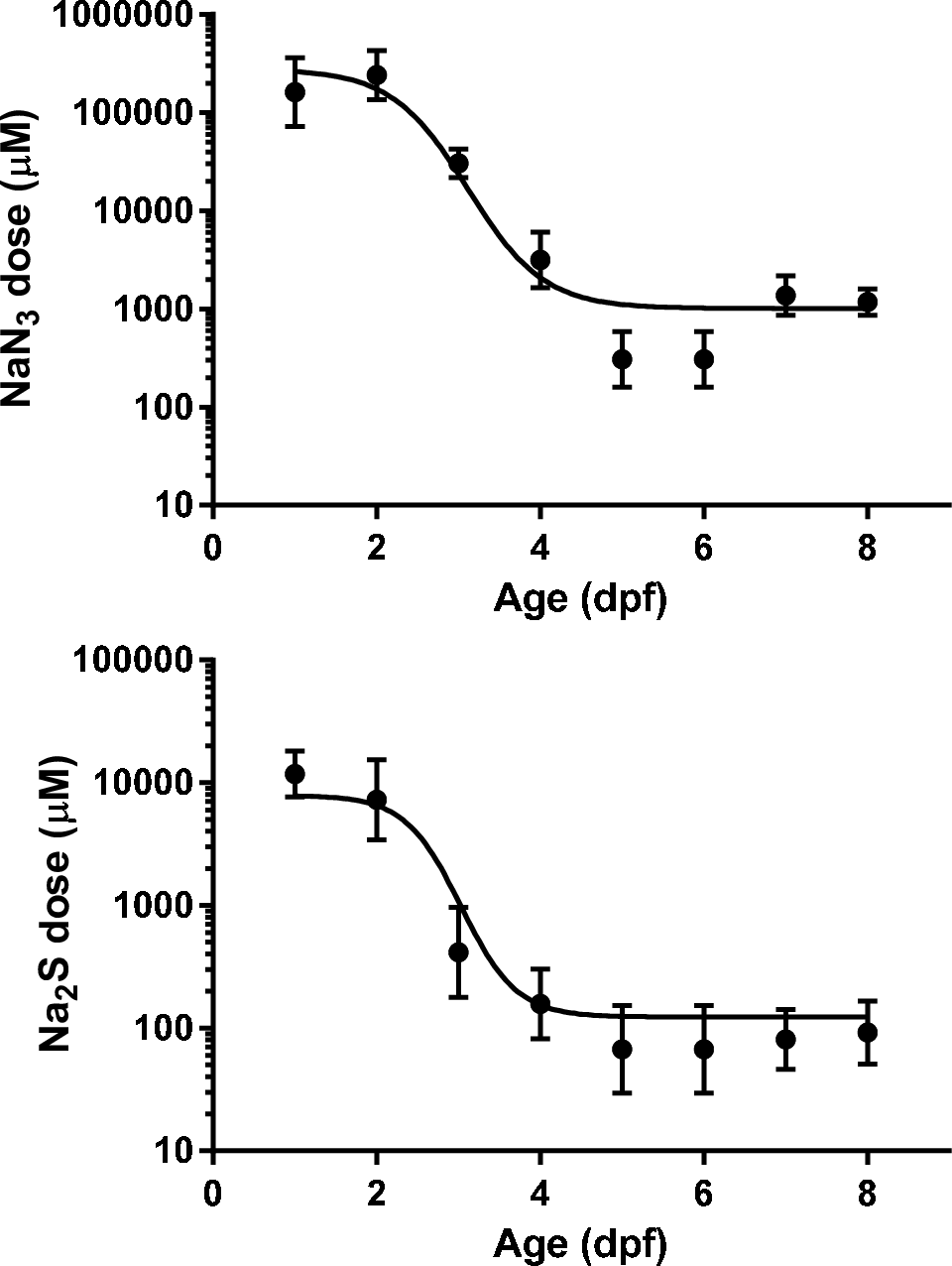

Supplement: S1 Fig — The LD50 for 3h exposure of zebrafish embryos and larvae to Na2S or NaN3 is shown as a function of the developmental age in days post fertilization (dpf). The best-fit sigmoidal curves, calculated by non-linear regression analysis, are plotted on the figure. (TIF) [file pone.0193889.s001.tif]
