# Supplementary material for: Identification of specific metabolic pathways as druggable targets regulating the sensitivity to cyanide poisoning
Source: PLoS One. 2018 Jun 7;13(6):e0193889. doi: 10.1371/journal.pone.0193889 (PMC5991913; doi:10.1371/journal.pone.0193889)
Supplement: S1 Table — Results of the pathway over-representation analysis of the metabolomics data comparing the KCN-induced metabolite response in 1 dpf embryos vs 7 dpf larvae using the online MetaboAnalyst 3.5 Metabolic pathway analysis module. The Danio rerio Kyoto Encyclopedia of Genes and Genomes (KEGG) identification number is given for each metabolic pathway. The total number of compounds, the random expected hits and the actually matched number from the user uploaded data (hits) are indicated for each pathway. The nominal p-value is calculated from the enrichment analysis, and the False Discovery Rate p is the p-value adjusted using the false discovery rate for the experiment. The Topology impact parameter is the pathway impact value calculated from pathway topology analysis. (DOCX) [file pone.0193889.s002.docx]

S1 Table. Metabolic pathways differently regulated between zebrafish of different age after exposure to cyanide.

| **Pathway** | | **KEGG pathway** | **Total compounds in pathway** | **Expected hits in pathway** | **Hits** | **Nominal p-value** | **False Discovery Rate p** | **Topology Impact** | |
| --- | --- | --- | --- | --- | --- | --- | --- | --- | --- |
| Citrate cycle (TCA cycle) | dre00020 | 20 | 0.73189 | 6 | 4.62E-05 | 0.0037453 | 0.3248 |  |  |
| Pyruvate metabolism | dre00620 | 22 | 0.80508 | 5 | 0.00087888 | 0.035595 | 0.18804 |  |  |
| Alanine, aspartate and glutamate metabolism | dre00250 | 24 | 0.87827 | 5 | 0.0013419 | 0.036232 | 0.18038 |  |  |
| Glycolysis or Gluconeogenesis | dre00010 | 26 | 0.95146 | 5 | 0.0019647 | 0.039785 | 0.20413 |  |  |
| Pyrimidine metabolism | dre00240 | 41 | 1.5004 | 6 | 0.0029767 | 0.048222 | 0.13862 |  |  |
| Butanoate metabolism | dre00650 | 22 | 0.80508 | 4 | 0.0071239 | 0.096172 | 0.10145 |  |  |
| Galactose metabolism | dre00052 | 26 | 0.95146 | 4 | 0.013069 | 0.15123 | 0.09737 |  |  |
| Pantothenate and CoA biosynthesis | dre00770 | 15 | 0.54892 | 3 | 0.015373 | 0.15565 | 0.03571 |  |  |
| Glyoxylate and dicarboxylate metabolism | dre00630 | 18 | 0.6587 | 3 | 0.025522 | 0.20673 | 0.14815 |  |  |
| Glycerolipid metabolism | dre00561 | 18 | 0.6587 | 3 | 0.025522 | 0.20673 | 0.41129 |  |  |
| Valine, leucine and isoleucine biosynthesis | dre00290 | 13 | 0.47573 | 2 | 0.079149 | 0.58282 | 0 |  |  |
| Ubiquinone and other terpenoid-quinone biosynthesis | dre00130 | 3 | 0.10978 | 1 | 0.10589 | 0.70817 | 1 |  |  |
| beta-Alanine metabolism | dre00410 | 16 | 0.58551 | 2 | 0.11366 | 0.70817 | 0.23256 |  |  |
| Phenylalanine, tyrosine and tryptophan biosynthesis | dre00400 | 4 | 0.14638 | 1 | 0.13868 | 0.80239 | 0 |  |  |
| D-Glutamine and D-glutamate metabolism | dre00471 | 5 | 0.18297 | 1 | 0.1703 | 0.86213 | 0 |  |  |
| Synthesis and degradation of ketone bodies | dre00072 | 5 | 0.18297 | 1 | 0.1703 | 0.86213 | 0.6 |  |  |
| Starch and sucrose metabolism | dre00500 | 22 | 0.80508 | 2 | 0.19095 | 0.90982 | 0.18942 |  |  |
| Arginine and proline metabolism | dre00330 | 43 | 1.5736 | 3 | 0.20573 | 0.9258 | 0.20479 |  |  |
| Glycerophospholipid metabolism | dre00564 | 28 | 1.0246 | 2 | 0.27338 | 1 | 0.16478 |  |  |
| Glycine, serine and threonine metabolism | dre00260 | 31 | 1.1344 | 2 | 0.31486 | 1 | 0 |  |  |
| Riboflavin metabolism | dre00740 | 11 | 0.40254 | 1 | 0.33745 | 1 | 0 |  |  |
| Amino sugar and nucleotide sugar metabolism | dre00520 | 37 | 1.354 | 2 | 0.39606 | 1 | 0.08058 |  |  |
| Nicotinate and nicotinamide metabolism | dre00760 | 14 | 0.51232 | 1 | 0.40817 | 1 | 0 |  |  |
| Histidine metabolism | dre00340 | 14 | 0.51232 | 1 | 0.40817 | 1 | 0.16667 |  |  |
| Valine, leucine and isoleucine degradation | dre00280 | 38 | 1.3906 | 2 | 0.40922 | 1 | 0.01219 |  |  |
| Tryptophan metabolism | dre00380 | 39 | 1.4272 | 2 | 0.42225 | 1 | 0.09711 |  |  |
| Pentose and glucuronate interconversions | dre00040 | 15 | 0.54892 | 1 | 0.43005 | 1 | 0 |  |  |
| Purine metabolism | dre00230 | 66 | 2.4152 | 3 | 0.43881 | 1 | 0.10668 |  |  |
| Tyrosine metabolism | dre00350 | 44 | 1.6102 | 2 | 0.48515 | 1 | 0.07069 |  |  |
| Propanoate metabolism | dre00640 | 20 | 0.73189 | 1 | 0.52813 | 1 | 0 |  |  |
| Fructose and mannose metabolism | dre00051 | 21 | 0.76848 | 1 | 0.54566 | 1 | 0 |  |  |
| Glutathione metabolism | dre00480 | 26 | 0.95146 | 1 | 0.62418 | 1 | 0 |  |  |
| Cysteine and methionine metabolism | dre00270 | 29 | 1.0612 | 1 | 0.66473 | 1 | 0.02332 |  |  |
| Fatty acid metabolism | dre01212 | 38 | 1.3906 | 1 | 0.76236 | 1 | 0 |  |  |
