# Supplementary material for: Identification of specific metabolic pathways as druggable targets regulating the sensitivity to cyanide poisoning
Source: PLoS One. 2018 Jun 7;13(6):e0193889. doi: 10.1371/journal.pone.0193889 (PMC5991913; doi:10.1371/journal.pone.0193889)
Supplement: S2 Table — Results of the pathway enrichment analysis of the metabolomics data comparing baseline metabolite concentration levels in 1 dpf embryos vs 7 dpf larvae using the online MetaboAnalyst 3.5 Metabolic pathway analysis module. Parameters shown are as in S1 Table. (DOCX) [file pone.0193889.s003.docx]

S2 Table. Differences in metabolic signature at baseline between 1 and 7 dpf zebrafish.

| **Pathway** | **KEGG pathway** | **Total compounds in pathway** | **Hits** | **Nominal p-value** | **False Discovery Rate p** | **Impact** |
| --- | --- | --- | --- | --- | --- | --- |
| Purine metabolism | dre00230 | 66 | 12 | 6.49E-11 | 2.53E-09 | 0.30886 |
| Pyrimidine metabolism | dre00240 | 41 | 12 | 1.49E-10 | 2.92E-09 | 0.56149 |
| Citrate cycle (TCA cycle) | dre00020 | 20 | 9 | 9.40E-08 | 7.54E-07 | 0.44332 |
| Glyoxylate and dicarboxylate metabolism | dre00630 | 18 | 5 | 9.46E-08 | 7.54E-07 | 0.5926 |
| beta-Alanine metabolism | dre00410 | 16 | 1 | 9.67E-08 | 7.54E-07 | 0 |
| Glycolysis or Gluconeogenesis | dre00010 | 26 | 5 | 1.75E-07 | 1.05E-06 | 0.20413 |
| Fructose and mannose metabolism | dre00051 | 21 | 2 | 2.24E-07 | 1.05E-06 | 0.00794 |
| Inositol phosphate metabolism | dre00562 | 27 | 1 | 2.25E-07 | 1.05E-06 | 0 |
| Amino sugar and nucleotide sugar metabolism | dre00520 | 37 | 5 | 2.43E-07 | 1.05E-06 | 0.12913 |
| Glycine, serine and threonine metabolism | dre00260 | 31 | 2 | 3.94E-07 | 1.54E-06 | 0 |
| Glycerolipid metabolism | dre00561 | 18 | 3 | 6.16E-07 | 2.04E-06 | 0.13031 |
| Glycerophospholipid metabolism | dre00564 | 28 | 3 | 6.27E-07 | 2.04E-06 | 0.19461 |
| Cysteine and methionine metabolism | dre00270 | 29 | 2 | 9.89E-07 | 2.97E-06 | 0.09586 |
| Lysine degradation | dre00310 | 18 | 1 | 1.58E-06 | 4.20E-06 | 0 |
| Nicotinate and nicotinamide metabolism | dre00760 | 14 | 3 | 1.73E-06 | 4.20E-06 | 0.18182 |
| Alanine, aspartate and glutamate metabolism | dre00250 | 24 | 5 | 1.86E-06 | 4.20E-06 | 0.16139 |
| Butanoate metabolism | dre00650 | 22 | 5 | 1.88E-06 | 4.20E-06 | 0.10145 |
| D-Glutamine and D-glutamate metabolism | dre00471 | 5 | 1 | 1.94E-06 | 4.20E-06 | 0 |
| Tyrosine metabolism | dre00350 | 44 | 3 | 2.15E-06 | 4.41E-06 | 0.07069 |
| Synthesis and degradation of ketone bodies | dre00072 | 5 | 1 | 2.30E-06 | 4.49E-06 | 0.6 |
| Taurine and hypotaurine metabolism | dre00430 | 7 | 1 | 2.66E-06 | 4.93E-06 | 0.2 |
| Pantothenate and CoA biosynthesis | dre00770 | 15 | 3 | 8.10E-06 | 1.44E-05 | 0 |
| Starch and sucrose metabolism | dre00500 | 22 | 3 | 1.44E-05 | 2.25E-05 | 0.29678 |
| Ascorbate and aldarate metabolism | dre00053 | 6 | 2 | 1.45E-05 | 2.25E-05 | 0.5 |
| Pentose and glucuronate interconversions | dre00040 | 15 | 2 | 1.45E-05 | 2.25E-05 | 0.58333 |
| Galactose metabolism | dre00052 | 26 | 4 | 2.23E-05 | 3.34E-05 | 0.04728 |
| Propanoate metabolism | dre00640 | 20 | 1 | 3.47E-05 | 5.02E-05 | 0 |
| Pyruvate metabolism | dre00620 | 22 | 5 | 0.001896 | 0.002641 | 0.18804 |
| Arginine and proline metabolism | dre00330 | 43 | 2 | 0.00337 | 0.004531 | 0.0331 |
| Porphyrin and chlorophyll metabolism | dre00860 | 27 | 1 | 0.00412 | 0.005355 | 0.0415 |
| Phenylalanine, tyrosine and tryptophan biosynthesis | dre00400 | 4 | 1 | 0.015247 | 0.018583 | 0 |
| Ubiquinone and other terpenoid-quinone biosynthesis | dre00130 | 3 | 1 | 0.015247 | 0.018583 | 1 |
| Glutathione metabolism | dre00480 | 26 | 5 | 0.025899 | 0.030608 | 0.45455 |
| Riboflavin metabolism | dre00740 | 11 | 1 | 0.077184 | 0.088535 | 0 |
| Folate biosynthesis | dre00790 | 16 | 1 | 0.1007 | 0.11221 | 0 |
| Tryptophan metabolism | dre00380 | 39 | 2 | 0.19276 | 0.20882 | 0.01766 |
| Terpenoid backbone biosynthesis | dre00900 | 15 | 1 | 0.76331 | 0.80457 | 0.14516 |
| Valine, leucine and isoleucine degradation | dre00280 | 38 | 3 | 0.82549 | 0.84721 | 0.02438 |
| Valine, leucine and isoleucine biosynthesis | dre00290 | 13 | 3 | 0.89706 | 0.89706 | 0 |
