# Supplementary material for: Identification of specific metabolic pathways as druggable targets regulating the sensitivity to cyanide poisoning
Source: PLoS One. 2018 Jun 7;13(6):e0193889. doi: 10.1371/journal.pone.0193889 (PMC5991913; doi:10.1371/journal.pone.0193889)
Supplement: S3 Table — EC50 doses calculated from 24h survival data of 7 dpf zebrafish larvae exposed to 20 μM KCN (S1 Dataset), as well as the EC50 dose for mortality in 1 dpf zebrafish embryos exposed to 500 μM KCN are shown, where applicable. Doses of the experimental drug (without KCN exposure) leading to zebrafish mortality after 24h exposure are shown as the relevant toxic dose of the drug. ND: not done; -: no effective dose found within range tested (the dose ranges went up to the maximum solubility of the respective drug in the embryo medium). (DOCX) [file pone.0193889.s004.docx]

S3 Table. Compounds tested in the focused zebrafish screen of metabolism regulating drugs.

|  | | | **Protective dose (24h survival) – EC50** | **Dose reversing resistance to KCN – IC50** | **Toxic dose (without KCN)** | |
| --- | --- | --- | --- | --- | --- | --- |
| **Drug name** | **PubChem ID** | **Putative mode of action** | **7 dpf fish + 20 µM KCN** | **1 dpf fish + 500 µM KCN** | **7 dpf fish** | **1 dpf fish** |
|  |  |  |  |  |  |  |
| Sodium glyoxylate | 23720423 | Substrate for malate synthase | 14 µM | ND | 100 mM | ND |
| Glyoxal trimer dihydrate | 92988 | Precursor of glyoxylate | 11 µM | ND | - | ND |
| Glyoxal solution | 7860 | Precursor of glyoxylate | 82 µM | ND | 100 mM | ND |
| Sodium pyruvate | 107735 | energy metabolism | 176 µM | ND | - | ND |
| α-ketoglutarate | 164533 | TCA metabolite | 354 µM | - | - | - |
| (±)-3-Methyl-2-oxovaleric acid sodium salt | 124203968 | α-ketoglutarate dehydrogenase inhibitor | 571 µM | ND | - | ND |
| Dihydroxyacetone | 670 | metabolite of glycolysis / cyanohydrin formation | 1 mM | ND | - | ND |
| Sodium dichloroacetate | 517326 | PDK inhibitor | 22 mM | ND | - | ND |
| UK-5099 | 6438504 | mitochondrial pyruvate carrier inhibitor | - | 25 µM | ND | 200 µM |
| Etomoxir | 23675376 | Carnitine palmitoyltransferase I inhibitor | ND | 35 µM | ND | 300 µM |
| Dimethyl succinate | 7820 | Succinate derivative | - | 408 µM | 310 µM | 5 mM |
| Sodium citrate | 23666341 | TCA metabolite | - | ND | 100 mM | ND |
| Sodium succinate | 124202561 | TCA metabolite | - | ND | - | ND |
| Oxaloacetate | 970 | TCA metabolite | - | ND | 10 mM | ND |
| Malate | 92824 | TCA metabolite | - | ND | 100 mM | ND |
| DL-Isocitric acid trisodium salt hydrate | 71306851 | TCA metabolite | - | ND | - | ND |
| Sodium fumarate dibasic | 6364607 | TCA metabolite | - | ND | 100 mM | ND |
| Cis-aconitic acid | 643757 | TCA metabolite | - | ND | 10 mM | ND |
| Dimethyl 2-oxoglutarate | 25775 | α-ketoglutarate derivative | - | - | 5 mM | 10 mM |
| Octyl-α-ketoglutarate | 11615849 | α-ketoglutarate derivative | - | - | 5 mM | - |
| Monomethyl hydrogen succinate | 77487 | succinate derivative | - | - | - | - |
| Sodium glutamate | 87090819 | α-ketoglutarate precursor | - | ND | 100 mM | ND |
| L-glutamine | 5961 | α-ketoglutarate precursor | - | ND | - | ND |
| Sodium L-lactate | 16219594 | Lactate dehydrogenase substrate | - | ND | 100 mM | - |
| AZD7545 | 16741245 | PDK inhibitor | - | ND | 1 mM | ND |
| Radicicol | 6323491 | PDK inhibitor | - | ND | 400 µM | ND |
| Leelamine hydrochloride | 16759156 | PDK inhibitor | - | ND | 20 µM | ND |
| Mito-dichloroacetate | N/A* | mitochondrially targeted PDK inhibitor | - | ND | 500 µM | ND |
| Ranolazine dihydrochloride | 71279 | pyruvate dehydrogenase activator | - | - | 5 mM | 5 mM |
| Insulin | N/A | glucose metabolism signaling hormone | - | ND | 1 mg/ml | - |
| CPI 613 | 24770514 | α-ketoglutarate dehydrogenase inhibitor | - | - | 62.5 µM | 62.5 µM |
| Sodium valproate | 16760703 | α-ketoglutarate dehydrogenase inhibitor | - | ND | 10 mM | ND |
| BPTES | 3372016 | glutaminase inhibitor | - | - | 250 µM | 250 µM |
| O-(Carboxymethyl)hydroxyl-amine hemihydrochloride | 2723609 | glutamate transaminase inhibitor | - | - | 5 mM | 2.5 mM |
| CB-839 | 71577426 | glutaminase inhibitor | - | - | 125 µM | 250 µM |
| 2-aminobicyclo (2.2.1) heptane 2-carboxylic acid | 44721409 | glutamate dehydrogenase activator | - | ND | - | ND |
| Dimethyl malonate | 7943 | succinate dehydrogenase inhibitor | - | - | 625 µM | 1.25 mM |
| Alexidine dihydrochloride | 102678 | succinate dehydrogenase activator | - | ND | 5 µM | ND |
| Oxalomalic acid sodium salt | 24941211 | isocitrate dehydrogenase inhibitor | ND | - | ND | - |
| Chlorothricin | 131668618 | malate dehydrogenase 2 inhibitor | - | - | - | - |
| Sodium oxamate | 71650611 | lactate dehydrogenase inhibitor | - | ND | - | ND |
| Bromopyruvic acid | 70684 | metabolic inhibitor | ND | - | ND | - |
| 2-deoxyglucose | 108223 | glycolysis inhibitor | ND | - | ND | - |
| Dorsomorphin | 11524144 | AMP kinase inhibitor | - | ND | 50 µM | - |
| Acadesine | 17513 | AMP kinase activator | - | ND | - | ND |
| 5-(Tetradecyloxy)-2-furoic acid | 115175 | Acetyl-CoA carboxylase inhibitor | - | ND | 100 µM | ND |
| 1,2,3-Benzenetricarboxylic acid hydrate | 16219028 | mitochondrial tricarboxylate carrier inhibitor | - | ND | 100 mM | ND |
| Dimethyloxalylglycine | 560326 | prolyl 4-hydrolyase inhibitor | - | ND | 1.25 mM | ND |
